# Migratory Polyarthritis in the Setting of Chlamydial Infection

**DOI:** 10.7759/cureus.5222

**Published:** 2019-07-24

**Authors:** Pooja Patel, Hitanshu Dave, Rupak Desai, Priyank J Yagnik, Elizabeth Davies

**Affiliations:** 1 Rheumatology, Advocate Aurora Health, Brookfield, USA; 2 Internal Medicine, Hackensack Meridian Health - Jersey Shore University Medical Center, Neptune City, USA; 3 Cardiology, Atlanta Veterans Affairs Medical Center, Decatur, USA; 4 Pediatrics, University of Kansas School of Medicine, Wichita, USA; 5 Family Medicine, Prohealth Medical Group, Waukesha, USA

**Keywords:** migratory, arthritis, oligoarthritis, reactive arthritis, arthralgia, infection, chlamydia trachomatis, case report, polyarthritis, reactive polyarthritis

## Abstract

Reactive arthritis is defined as a sterile inflammation involving the synovial membrane, tendons and/or fascia, elicited by an infection, usually originating from gastrointestinal or genitourinary tracts. Reactive arthritis can also be triggered by a sexually transmitted disease, referred to as sexually acquired reactive arthritis. The most common identifiable cause of non-gonococcal urethritis is *Chlamydia trachomatis*. Herein, we present a case of a 30-year-old healthy male patient, who developed migratory oligoarthritis in the setting of elevated inflammatory markers, highlighting the importance of obtaining an appropriate history and ordering pertinent laboratory tests, along with literature review on reactive arthritis.

## Introduction

Reactive arthritis is a rare cause of inflammatory arthritis that occurs following specific genitourinary and gastrointestinal infections: *Chlamydia trachomatis* and *Ureaplasma urealyticum* in the urethra and *Campylobacter*, *Escherichia coli*, *Salmonella*, *Shigella* and *Yersinia *in the intestine. The term “reactive arthritis” was coined in 1969. It was defined as an “arthritis” that develops during or after an infection elsewhere in the body, but in which a microorganism does not enter the joint cavity [1]. Therefore, the term reactive arthritis was suggested to be more accurate for the entire spectrum of arthritis, triggered by a distant infection (may or may not be detected in the joint) or irrespective of the human leukocyte antigen (HLA)-B27 association [2].

## Case presentation

A 30-year-old healthy male patient presented to the emergency department with chief concerns of new-onset lower extremity and lower back pain. The patient’s symptoms began about two weeks ago, with discomforting pain in his left groin that has progressively worsened. The patient complained of pain in both his thighs, knees, hip and lower back. The patient denied any triggering events: heavy lifting, pushing, or pulling. He denied any recent trauma, injury or accident; any fever, chills, night sweats, nausea or vomiting; any bowel/bladder incontinence or saddle anesthesia; any new-onset weakness, tingling or numbness in his extremities. He denied taking any prescription medications or using any illicit drugs. He also denied any family history of autoimmune disorders.

On physical examination, the patient was afebrile and well appearing. The patient was mildly tachycardic. On examining his lower extremities, the patient had pain mainly in his hamstring and thigh muscles, worse on the left. The patient had mild pain in bilateral paraspinal muscles without any significant mid-line vertebral pain. His straight leg raise test was negative. Strength in all muscle groups scored 5 on a scale of 0 to 5, sensory and motor functions scored 5 on a scale of 0 to 5 and deep tendon reflexes were normal. The patient’s lower extremities and skin were warm and well perfused with normal peripheral pulses. Laboratory tests were obtained as listed in Table 1.

**Table 1:** Laboratory Test Results

Component	Reference Range	Day 1
Hematology:		
White blood cell count	4.0-10.8 kilo per microliter	7.43
Red blood cell count	4.5-6.1 million per microliter	4.81
Hemoglobin	14.0-17.0 gram per deciliter	13.1 (low)
Hematocrit	42.0-52.0 percentage	39.5 (low)
Mean corpuscular volume	81-99 femtoliter	82.1
Mean corpuscular hemoglobin	27-33 picogram	27.2
Mean corpuscular hemoglobin concentration	32-36 gram per deciliter	33.2
Platelet count	130-400 kilo per microliter	210
Red cell distribution width	35-46 femtoliter	36.4
Mean platelet volume	9.4-12.4 femtoliter	11.0
Basic metabolic panel:		
Sodium	137-146 millimole per liter	134 (low)
Potassium	3.6-5.0 millimole per liter	3.6
Chloride	98-112 millimole per liter	99
Carbon dioxide	21-32 millimole per liter	28
Calcium	8.4-10.2 milligram per deciliter	8.7
Creatinine	0.70-1.20 milligram per deciliter	0.90
Blood urea nitrogen	8-25 milligram per deciliter	16
Glucose	70-99 milligram per deciliter	94
Estimated glomerular filtration rate, non-African American	>59.9 milliliter per minute per 1.73 square meter	114
Estimated glomerular filtration rate, African American	>59.9 milliliter per minute per 1.73 square meter	>120
Anion gap	3.00-14.00 millimole per liter	7
Inflammatory markers:		
Sedimentation rate	0-10 millimeter per hour	54 (high)
C-reactive protein	0-1.0 milligram per deciliter	12.80 (high)
Creatine kinase, total	50-308 units per liter	82
Blood culture:		
Specimen description	Blood	
Culture	No growth 5 days	

The patient was given ketorolac, a nonsteroidal anti-inflammatory drug (NSAID), in addition to intravenous methylprednisolone, providing him moderate symptomatic relief. The patient was discharged home with oral methylprednisolone (Medrol Dosepak) in a stable condition with a clinical diagnosis of myalgia. The patient was ruled out of having polymyositis or rhabdomyolysis in the setting of a normal creatine kinase level, infectious arthritis due to normal white cell count.

The patient followed up at the clinic a few days later, for concerns of migratory polyarthritis. The patient complained of ongoing left hip pain, describing it as sharp in nature and associated with muscle tightness. The patient reported intermittent, waxing and waning, but progressively worsening joint pain and stiffness in his lower back and spine, and right shoulder. He rated his pain as 6-8 on a scale of 1 to 10. The patient also complained of left knee pain with associated mild to moderate swelling, affecting his ability to walk. The patient denied any symptomatic improvement with the Medrol Dosepak prescribed to him in the emergency department. The patient was taking occasional NSAIDs for pain relief, providing him “some” relief. The patient enjoys hiking but denied any recent exposure to ticks. The patient has been sexually active with female partners but denied any new sexual partners in the past few months. The patient denied any past medical history of sexually transmitted diseases. On review of systems, the patient reported subjective low-grade fever without chills, malaise, night sweats or weight loss. He reported mild dysuria in the last one week, without hematuria. The patient had an unremarkable physical examination except for 1+ swelling of his left knee without increased warmth or erythema, and moderately decreased passive range of motion. Under sterile conditions, an injection of a 40-cubic centimeter​​​ of Kenalog-40 was administered in his left knee for pain relief.

**Figure 1:**
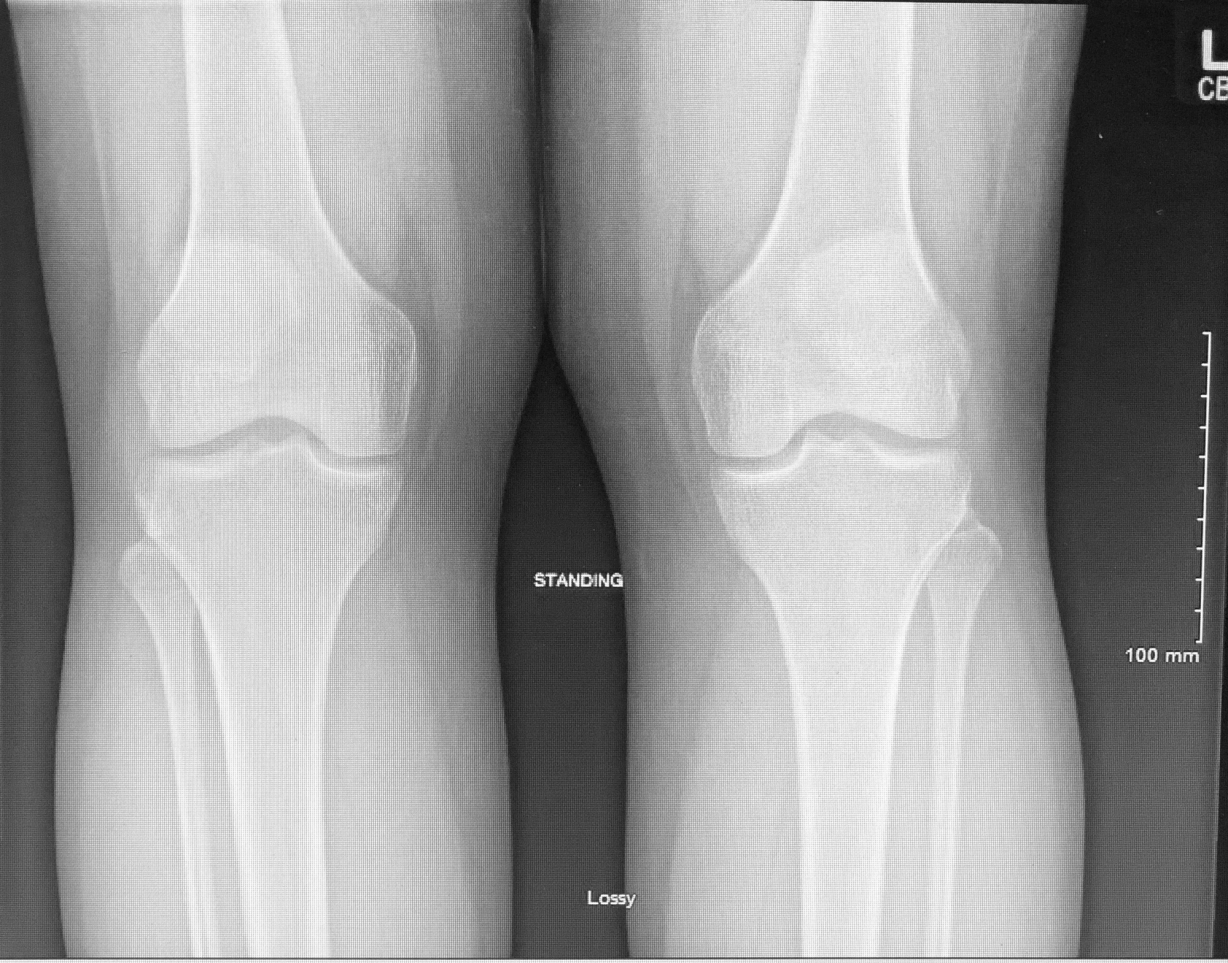
X-ray Bilateral Knee in Standing View

Laboratory tests were ordered for antinuclear antibody (ANA) screen, thyroid-stimulating hormone (TSH), rheumatoid factor (RF), anti-cyclic citrulline peptide (anti-CCP) antibody and urinalysis with microscopy; all had normal results. The patient had a negative ANA result as well as normal TSH level at 1.52 microliter per milliliter (normal 0.35-4.94 microliter per milliliter), RF was <10 units per milliliter (normal <15 units per milliliter) and anti-CCP antibody was 8 millimole per liter (normal 3.00-14.00 millimole per liter) and a normal urinalysis. An x-ray of bilateral knee demonstrated no acute fracture or dislocation. Medial, lateral and patellofemoral compartments were well maintained. No acute osseous findings were noted (Figure 1).

The patient was recommended to continue taking ibuprofen 600 mg three times daily with food, regularly for at least 14 days. He was advised to wear a knee brace for support while physically active and elevate his legs while resting. Further laboratory tests were ordered (Table 2).

**Table 2:** Laboratory Test Results N/A, not applicable

Diagnostic procedure	Result	Units	Reference range
Sedimentation rate	33 (high)	millimeter per hour	0-10
Hepatitis function panel			
Albumin	3.5	gram per deciliter	3.4-5.0
Total bilirubin	0.3	milligram per deciliter	0.2-1.4
Bilirubin, direct	<0.1	milligram per deciliter	0.0-0.2
Alkaline phosphatase	109	units per liter	38-127
Aspartate aminotransferase/serum glutamic-oxaloacetic transaminase	19	units per liter	9-40
Alanine aminotransferase/serum glutamic-pyruvic transaminase	50	units per liter	12-64
Total protein	8.6 (high)	gram per deciliter	6.4-8.2
Human immunodeficiency virus 1/human immunodeficiency virus 2 antibody, protein 24 antigen	Non-reactive	N/A	Non-reactive
Parvovirus B19 antibody, immunoglobulin G and immunoglobulin M:			
Parvovirus immunoglobulin G	3.7 (high)		
	Unit: index value 0.9 index value: negative- no significant level of detectable parvovirus B19 immunoglobulin G antibody 0.9-1.1 index value: equivocal – repeat testing in 10-14 days may be helpful >1.1 index value: positive- immunoglobulin G antibody to parvovirus B19 detected, which may indicate a current or past infection		
Parvovirus immunoglobulin M	0.2		
	Unit: index value <0.9 index value: negative- no significant level of detectable parvovirus B19 immunoglobulin M antibody 0.9-1.1 index value: equivocal – repeat testing in 10-14 days may be helpful >1.1 index value: positive- immunoglobulin M antibody to parvovirus B19 detected, which may indicate a current or past infection reference range: <0.9		
Chlamydia/Gonorrhea by nucleic acid amplification source: urine			
Chlamydia trachomatis	Positive	Positive results are reported to the State Department of Public Health Reference range: negative	
Neisseria gonorrhea	Negative		

The patient was informed of the laboratory test results and was started on doxycycline 100 mg twice daily for seven days. The patient was encouraged to contact his sexual partner(s) and recommend treatment. He was advised to avoid sexual activity until the treatment was completed and educated about the importance of using condoms (barrier protection). The State Department of Public Health was contacted and informed.

## Discussion

Reactive arthritis is a relatively rare disease that typically occurs in young adults, affecting both men and women. In a population-based study, the incidence of reactive arthritis following documented enteric bacterial infections ranged from 0.6 to 3.1 cases per 100,000, depending on the organism [3]. The causative pathogens, incidence and prevalence of reactive arthritis depend on the geographic region. In general, among the pathogens, *Chlamydia *is probably the most endemic. A systematic literature review conducted in 2016 identified only three studies of low to moderate quality reporting an incidence of sexually acquired reactive arthritis of 3.0 to 8.1 percentage [4].

The onset of reactive arthritis is usually acute or sporadic. Patients usually develop symptoms two to four weeks from the start of infection to the onset of musculoskeletal symptoms; in *Chlamydia *infection, the interval can extend up to four weeks. The disease is uncommon in children. Male and female patients are equally at risk for developing reactive arthritis induced by gastrointestinal infection, while male patients are more frequently diagnosed with reactive arthritis triggered by *Chlamydia trachomatis* infection [5].

Patients have typically asymmetric oligoarthritis, often in large joints of lower extremities. Patients also have arthritis in their upper extremities. Occasionally, a mild polyarticular form of arthritis, particularly in the small joints, can occur. Patients can also have dactylitis (sausage digit). In addition to arthritis, patients can develop bursitis, enthesitis, and tendonitis. Other extra-articular features include inflammatory back pain, eye disease; conjunctivitis (more common), anterior uveitis, skin changes; erythema nodosum, keratoderma blenorrhagicum, and circinate balanitis. Circinate balanitis has most frequently been associated with *Chlamydia arthritis*.

The diagnosis of reactive arthritis relies on the typical clinical presentation and the triggering infection. During the earlier symptomatic phase of enteric infections, isolation is usually possible from the stools. Hence, the diagnosis of reactive arthritis is often dependent on the detection of specific antibodies in the serum (Tables 3, 4) [5-7].

**Table 3:** Detection of Specific Antibodies

	Antibody class found positive:	Interpretation:
Antibody constellations which are not sufficient for the diagnosis of reactive arthritis	Only immunoglobulin G	Indicates former contact/infection, of limited value in reactive arthritis
	Only immunoglobulin A	Rare, may eventually occur in persisting infection
	Only immunoglobulin M	Of very limited value in reactive arthritis, control serology is recommended after four to six weeks
Typical antibody constellations in reactive arthritis	Immunoglobulin G and immunoglobulin M	Characteristic for active or recent infection
	Immunoglobulin G and immunoglobulin A	Characteristic for active, persisting or recent infection

**Table 4:** Serology to Detect Specific Organisms "+", positive "-", negative

Diagnosis	Serology:	Detection of the organism:
Post-urogenital reactive arthritis:		
Chlamydia trachomatis	+	Polymerase chain reaction in first-void urine
Mycoplasma	-	Culture from urogenital swab
Post-enteric reactive arthritis:		
Yersinia	+	Stool culture
Salmonella	+	Stool culture
Campylobacter	+	Stool culture
Shigella	-	Stool culture

Acute-phase reactants like erythrocyte sedimentation rate and/or C-reactive protein may be elevated. However, these may be normal in many patients. Studies have reported 60 to 80 percentage of HLA-B27-positive patients with reactive arthritis. Synovial fluid findings are nonspecific and are characteristic of inflammatory arthritis, with elevated leukocyte counts, predominantly neutrophils. Synovial fluid leukocyte count is between 2,000 and 64,000 millions per cubic millimeter [6]. There are no specific findings on plain radiographs that can establish the diagnosis of reactive arthritis. In patients with chronic joint disease, imaging studies such as ultrasonography, scintigraphy (radionuclide bone scanning) or magnetic resonance imaging can also identify changes consistent with peripheral synovitis, enthesitis or sacroiliitis.

Reactive arthritis is a clinical diagnosis based on the pattern of findings and exclusion of other diseases. There is no single definitive diagnostic test, nor are there validated diagnostic criteria.

Acute inflammatory monoarthritis or oligoarthritis may occur in a variety of disorders. The differential diagnosis of reactive arthritis can be guided, in part, by the pattern of symptoms and findings that are associated with arthritis and that may suggest a related infectious or other systemic disorder. The following differential diagnosis should be considered (Table 5).

**Table 5:** Differential Diagnosis N/A,  not applicable

Pattern of symptoms:	Differential diagnosis:	Confirmatory test:	Points to note:
Monoarthritis	Traumatic arthritis	Imaging study example. x-ray of the joint	Inquire about recent history of trauma or injury
	Gout	Serum uric acid level, inflammatory markers, synovial fluid examination; for crystal study (needle-shaped monosodium urate crystals which are “negatively birefringent”) and cell study	Personal past medical history of gout, family history of gout
	Calcium pyrophosphate deposition disease	Inflammatory markers, synovial fluid examination; for crystal study (rhomboid or rectangular shaped, “positively birefringent”) and cell study, and imaging study example. x-ray of the joint suggestive of chondrocalcinosis	Usually seen in the elderly population
	Septic arthritis	Synovial fluid examination (suggestive of leukocytosis, low glucose level, high protein level), inflammatory markers	History of recent trauma or injury, cellulitis-like symptoms
	Lyme’s disease	Lyme disease antibody screen	History of deer tick exposure
Oligoarthritis	Parvovirus infection	Parvovirus serology tests, inflammatory markers	Recent history of flu-like symptoms/viral syndrome symptoms
Diarrhea and arthritis	Enteroviral infection	N/A	Usually self-limited
	Bacterial infection	Stool examination with culture or serology levels	N/A
	Autoimmune etiology of diarrhea	Antinuclear antibody screen with cascade, specific antibody screen, biopsy or culture	Detailed history of symptoms, disease-specific antibody screen
Genitourinary symptoms and arthritis	Disseminated gonococcal infection	Urethral or cervical swabs, urinary gonococcal and chlamydial screen, nucleic acid amplification test, synovial fluid examination	Detailed history of urinary tract infection symptoms, past medical history of sexually transmitted diseases, sexual history
	Chlamydia trachomatis		

The management of the patient depends on the triggering factor contributing to their symptoms. Hence, there are several major aspects of its management. Antibiotics are not used to treat arthritis specifically; however, it may be indicated for the treatment of the underlying infection if there is evidence of ongoing genitourinary infection or gastrointestinal infection or carriage of potentially pathogenic organisms. In patients with chronic reactive arthritis induced by enteric bacteria, the available evidence does not support the use of long-term antibiotics [8, 9]. The main objectives of therapeutic management in a patient with reactive arthritis are patient education and pain relief to facilitate physical therapy. Educating the patient regarding the good prognosis is of utmost importance, to resolve their fears of serious physical impairment in the future.

Antibiotic use to treat ne*w *urogenital tract infections can substantially decrease the risk of reactive arthritis*. Chlamydia trachomatis *and *Neisseria gonorrhea* are venereal diseases; hence, treatment is of utmost value even in the absence of arthritis. *Chlamydia trachomatis *is treated with azithromycin (1 g orally as a single dose) or doxycycline (100 mg twice daily for seven days). The patient’s sexual partner(s) should simultaneously be treated to prevent reinfection. *Neisseria gonorrhea* should be treated with a single dose of 250 mg of intramuscular ceftriaxone and 1 g of oral azithromycin. There are no data to show that treatment of diarrhea with antibiotics has an impact on possible subsequent arthritis, and such treatment is therefore not recommended [7]. Controlled studies have shown no beneficial effects favoring the prolonged long-term use of antibiotics for the treatment of reactive arthritis except lymecycline showed to reduce the duration of *Chlamydia*-induced arthritis in a one-year study. This promising result was not confirmed either in two subsequent studies using tetracycline or in other trials comparing ciprofloxacin or azithromycin with placebo [7]. 

To treat acute arthritis, the use of non-steroidal anti-inflammatory drugs, often in high dose, is usually of major benefit. Intra-articular glucocorticoid injections can be administered if the patient has mono- or oligoarticular joint disease. Enthesopathy responds to local glucocorticoid injections.

Systemic glucocorticoids are indicated in patients with severe polyarthritis, high systemic inflammation, febrile patient or patient with symptoms of carditis or atrioventricular conduction disturbance. The starting dose of prednisone/prednisolone is usually 20-40 milligram per day [5]. If symptoms persist beyond three to six months, the use of disease-modifying anti-rheumatic drugs such as sulfasalazine, methotrexate or tumor necrosis factor alpha inhibitors may be necessary for symptom control and to prevent joint erosion. Therapy is discontinued three to six months following disease remission.

The course of reactive arthritis is variable, depending upon the triggering pathogen and the genetic background of the host. The typical disease duration is three to five months. Most patients either remit completely or have little active disease within six to 12 months after presentation, but 15% to 20% may experience more chronic persistent arthritis. After entering remission of peripheral joint arthritis, pain is occasionally still noted in the joints, at enthesitis or in the spine [5, 6].

## Conclusions

Reactive arthritis has been defined by consensus as a form of arthritis that is associated with a coexisting or recent antecedent extra-articular infection. It thus represents an excellent example for acquiring a detailed history of presenting illness, past medical history, social and family history, and understanding pathophysiology, improving diagnosis, and developing etiology-based treatment strategies. The goal of treatment for reactive arthritis is to treat the underlying cause of symptoms, eliminate the causative organism persisting in the host, and provide patient symptomatic relief.
